# Correction: Suzuki et al. Cross-Country Student Perceptions about Online Medical Education during the COVID-19 Pandemic. *Int. J. Environ. Res. Public Health* 2022, *19*, 2840

**DOI:** 10.3390/ijerph20075330

**Published:** 2023-03-30

**Authors:** Tomoya Suzuki, Anju Murayama, Yasuhiro Kotera, Divya Bhandari, Yuki Senoo, Yuta Tani, Kayo Harada, Ayumu Kawamoto, Satomi Sato, Toyoaki Sawano, Yasushi Miyata, Masaharu Tsubokura, Tetsuya Tanimoto, Akihiko Ozaki

**Affiliations:** 1 Medical Governance Research Institute, Takanawa, Minato-ku, Tokyo 1087505, Japan; 2 School of Medicine, Akita University, 1-1-1 Hondo, Akita 0108543, Japan; 3 School of Health Sciences, University of Nottingham, Nottingham NG7 2HA, UK; 4 Department of Surgery, Jyoban Hospital of Tokiwa Foundation, Iwaki 9728322, Japan; 5 Department of Primary Care and Community Health, Aichi Medical University, Nagakute 4801195, Japan; 6 Department of Radiation Health Management, Fukushima Medical University School of Medicine, Fukushima 9601247, Japan; 7 Department of Internal Medicine, Navitas Clinic Kaswasaki, Kawasaki 2100007, Japan; 8 Department of Breast Surgery, Jyoban Hospital of Tokiwa Foundation, Iwaki 9728322, Japan

## Additional Affiliation

In the published publication, there was an error regarding the affiliation for “Tomoya Suzuki”. In addition to affiliation “2” should be “School of Medicine, Akita University, 1-1-1 Hondo, Akita 0108543, Japan”. The authors apologize for any inconvenience caused and state that the scientific conclusions are unaffected. The original publication has also been updated [1].
